# Gut Microbiota Metabolites in NAFLD Pathogenesis and Therapeutic Implications

**DOI:** 10.3390/ijms21155214

**Published:** 2020-07-23

**Authors:** Jiezhong Chen, Luis Vitetta

**Affiliations:** 1 Medlab Clinical, Sydney 2015, Australia; 2 Faculty of Medicine and Health, The University of Sydney, Sydney 2006, Australia

**Keywords:** butyrate, bile acids, PXR, TGR5, NAFLD, probiotics, prebiotics, synbiotics, FMT, FMD

## Abstract

Gut microbiota dysregulation plays a key role in the pathogenesis of nonalcoholic fatty liver disease (NAFLD) through its metabolites. Therefore, the restoration of the gut microbiota and supplementation with commensal bacterial metabolites can be of therapeutic benefit against the disease. In this review, we summarize the roles of various bacterial metabolites in the pathogenesis of NAFLD and their therapeutic implications. The gut microbiota dysregulation is a feature of NAFLD, and the signatures of gut microbiota are associated with the severity of the disease through altered bacterial metabolites. Disturbance of bile acid metabolism leads to underactivation of bile acid receptors FXR and TGR5, causal for decreased energy expenditure, increased lipogenesis, increased bile acid synthesis and increased macrophage activity. Decreased production of butyrate results in increased intestinal inflammation, increased gut permeability, endotoxemia and systemic inflammation. Dysregulation of amino acids and choline also contributes to lipid accumulation and to a chronic inflammatory status. In some NAFLD patients, overproduction of ethanol produced by bacteria is responsible for hepatic inflammation. Many approaches including probiotics, prebiotics, synbiotics, faecal microbiome transplantation and a fasting-mimicking diet have been applied to restore the gut microbiota for the improvement of NAFLD.

## 1. Introduction

Nonalcoholic fatty liver disease (NAFLD) is defined as more than 5% fat accumulation in hepatocytes [1]. It is a common disease, accounting for one-third of the population in developed countries. NAFLD is closely associated with type 2 diabetes and other metabolic abnormalities including obesity, hypercholesterolemia and insulin resistance [2]. NAFLD can progress from simple hepatic steatosis to steatohepatitis (NASH) which is characterized by inflammation and hepatocyte degeneration. A small proportion of NASH will further progress to liver fibrosis/cirrhosis and hepatocellular carcinomas. At present, no effective therapeutic agents have been approved by FDA for the treatment of the disease. Weight loss has been considered as an effective approach; and 10% weight loss is sufficient to alleviate the disease [1]. However, most NAFLD patients are not able to achieve such weight loss. Therefore, the requisite is the investigation of other effective therapeutic approaches.

Gut microbiota has often been linked with metabolic diseases such as NAFLD, diabetes and obesity [3,4]. Many studies have shown that dysregulation of the intestinal microbiota (gut dysbiosis) can lead to NAFLD [5,6,7]. Therefore, modulation of the gut microbiota has been extensively studied for the treatment of the disease. Moreover, studies have demonstrated that several commensal bacterial metabolites can mediate the effects of the gut microbiota on the host and that their changes are involved in the pathogenesis of NAFLD [5,6,7]. Various therapeutic approaches for improving the diversity and abundance of the gut microbiota such as the inclusion of probiotics, prebiotics, synbiotics, faecal microbiome transplantation (FMT) and fasting-mimicking diet (FMD) could be advanced. In this review, we summarize and further discuss the mechanisms that the intestinal microbiota could affect the pathogenesis of NAFLD with the emphasis being on bacterial metabolites and approaches that beneficially modulate the intestinal microbiota to improve NAFLD.

## 2. Evidence of the Importance of the Intestinal Microbiota in NAFLD

Changes in abundance and diversity of the gut microbiota that have linked it to the progression of NAFLD have been characterized. Each stage of NAFLD has a special gut microbiota signature [5]. The severity of NAFLD has been associated with dysbiosis and loss of commensal bacterial metabolic functions [5,8,9]. In NAFLD, at the bacterial phylum level, Bacteroidetes are reported decreased, while levels of Firmicutes and Proteobacteria are increased [5]. At the bacterial family level, *Enterobacteriaceae* has been reported increased while *Rikenellaceae* and *Ruminococcaceae* are decreased. At the bacterial genera level, *Escherichia*, *Dorea*, *Peptoniphilus* are reported increased and *Anaerosporobacter*, *Coprococcus*, *Eubacterium*, *Faecalibacterium* and *Prevotella* are decreased [5]. Changes in microbiota in NASH are reported overlapped with steatosis with differences identified, especially those diagnosed with NASH with fibrosis. For example, *Eubacterium rectale* is increased in moderately severe NAFLD but decreased in NASH with fibrosis. Compared to steatosis, the gut microbiome associated with NASH presents with a higher abundance of Bacteroides than that with fibrosis that presents with significantly increased *Ruminococcus* [8]. Schwimmer et al. (2019) studied the gut microbiota in NAFLD children, reporting that NAFLD had lower alpha-diversity of gut microbiota compared with healthy controls, while NASH had the lowest alpha-diversity [10]. The expression of genes involved in lipopolysaccharides (LPS) synthesis in gut microbiota was increased in NASH compared with steatosis while increased flagellar biosynthesis gene expression in NASH indicated fibrosis. Furthermore, bacterial translocation due to increased gut permeability and increased blood levels of LPS have been associated with NAFLD [11,12]. 

The important role of the gut microbiota in NAFLD has been demonstrated in murine models. Several studies have shown that FMT of germ-free mice from NAFLD mice presents partial NAFLD histology. Roy et al. (2013) used high-fat diet (HFD) feeding to establish two mouse models [13]. One had weight gain with hyperglycaemia and high systemic pro-inflammatory cytokines, whereas the other had similar weight gain but with normoglycaemia and low pro-inflammatory cytokines. FMT from the two types of mice to germ-free mice produced two distinguishable phenotypes, with and without hepatic steatosis and increased lipogenesis. The two phenotypes also had a distinguishable gut microbiota, suggesting that the determining effect was due to the gut microbiota. Soderborg et al. (2018) did FMT to germ-free mice from 2-week-old infants of obese and lean mothers, respectively [14]. The mice with a transplanted faecal microbiome from obese mothers born mice showed NAFLD-like historical changes with periportal inflammation. 

Furthermore, experiments with FMTs from NAFLD patients to germ-free mice have vilified the role of the gut microbiota in the pathogenesis of NAFLD. Chiu et al. (2017) transferred the gut microbiome from NASH patients to germ-free mice while feeding the animals with an HFD [15]. The mice had increased epididymal fat weight, hepatic steatosis, inflammation and multifocal necrosis. There were elevated levels of serum ALT, AST, endotoxin, IL-6 and Mcp1. The mRNA levels of TLR2, TLR4, TNF-alpha, Mcp1 and PPAR-gamma were also markedly increased. In contrast, germ-free mice fed with an HFD only had lipid accumulation and minor liver inflammation. Hoyles et al. (2018) also demonstrated that microbiome transplantation from NAFLD patients to germ-free mice resulted in hepatic steatosis and a NAFLD gut microbiota signature [16]. 

## 3. Gut Microbiota Metabolites in the Pathogenesis of NAFLD

In healthy conditions, the lumen of the intestines is the locale of an extensive network of commensal bacterial niches. The potential contribution effects of the gut microbiota on extraintestinal organs are achieved by various bacterial metabolites such as bile acids, short-chain fatty acids, amino acids, choline and ethanol. In NAFLD, these metabolites are altered and involved in the pathogenesis of NAFLD. 

### 3.1. Bile Acid Alterations

Bile acids have been recognized to not only facilitate fat digestion and absorption but also play key roles in many physiological processes. Bile acids are important signaling molecules, regulating bile acid synthesis and energy balance [17,18]. The size and composition of the bile acid pool in the enterohepatic circulation is sophisticatedly regulated through their hepatic biosynthesis and metabolism by the gut microbiota. The interactions of bile acids and the gut microbiota are bidirectional [18]. The gut microbiota plays a key role in the metabolism of bile acids, converting primary bile acids into secondary bile acids. Thus, intestinal dysbiosis can lead to deficits in bile acids, which can in turn affect the gut microbiota composition and energy balance. 

Numerous reactions are involved in the conversion of bile acids by the gut microbiota including deconjugation, 7alpha-dehydroxylation, esterification, desulphication and oxidation/epimerization [18]. The bile acids are deconjugated by bile salt hydrolases (BSHs), which have been identified in intestinal bacteria that belong to the genera *Bacteroides*, *Bifidobacterium*, *Clostridium*, *Lactobacillus* and *Listeria* [19]. The deconjugated primary bile acids are then processed by 7alpha-dehydroxylase into secondary bile acids. The enzyme is mainly synthesized from *Clostridium* and *Eubacterium* species from the Firmicutes phylum [19]. Through oxidation/epimerization, bile acids are metabolized into beneficial bile salts such as ursodeoxycholic acid. Bile acid excretion through the faeces is facilitated through chemical esterification and desulphication processes. 

In NAFLD, the abundance of bacteria that convert primary bile acids into secondary bile acids is decreased. This leads to not only decreased stimulation of bile acid receptors by secondary bile acids but also further disturbance of the gut microbiota [18]. The composition of the bile acid pool is important to maintain commensal bacterial community diversity. The detergent effect of bile acids can inhibit certain types of bacteria but not others so that the balanced growth of various commensal bacteria is well maintained. Chen et al. (2019) found that the conjugated chenodeoxycholic acid/muricholic acid ratio was associated with NASH severity in 134 human NAFLD subjects [20]. Decreased deconjugation could also result in decreased production of taurine. A recent study showed the beneficial effect of taurine on hepatic steatosis and inflammation in a NAFLD murine model, which was established by FXR knockout [21]. Supplementation of the mice with 0.5% taurine in the drinking water increased hepatic but not blood taurine concentrations. Taurine reduced hepatic triglycerides, nonesterified fatty acids and total bile acids in FXR knockout mice with decreased expression of fatty acid synthetic genes *Acc1* and *Scd1* as well as decreased oxidative stress-related genes *Hmox1* and *Gsta1*. 

**Figure 1:**
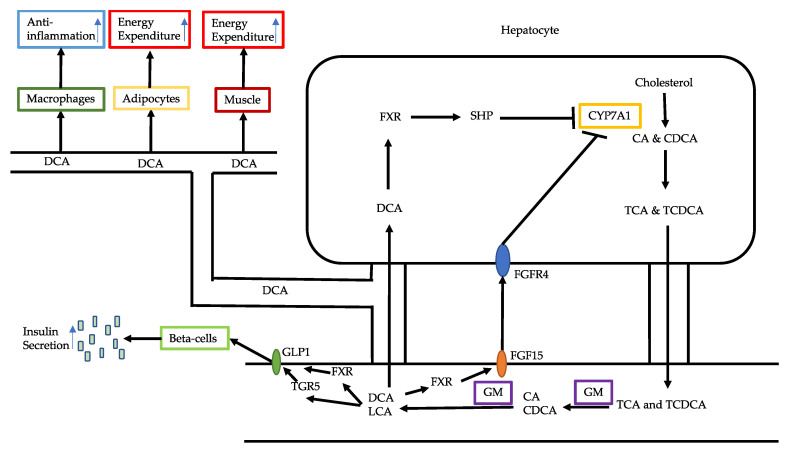
Bile acids and their receptors in NAFLD. Cholesterol is converted by CYP7A1 into primary bile acids CA and CDCA, which are conjugated and secreted into the intestines. In the intestines, TCA and TCDCA are deconjugated and converted into secondary bile acids DCA and LCA by GM. DCA and LCA stimulates the secretion of intestinal FGF15, which circulates to the liver and binds to FGFR4, causing inhibition of CYP7A1. DCA also circulates into the liver to activate PXR in the liver, which leads to inhibition of SHP, an inhibitor of CYP7A1. DCA stimulates the secretion of intestinal GLP-1 through activation of PXR and TGR5. GLP-1 binds to GLP-1R on beta-cells to stimulate insulin secretion. DCA in circulation can also act on macrophages to exert anti-inflammatory effect and act on adipocytes and muscle cells to increase energy expenditure. Abbreviations: CYP7A1, cytochrome P450 family 7 subfamily A member 1; CA, cholic acid; CDCA, chenocholic acid; TCA, taurocholic acid; TCDCA, taurochenocholic acid; DCA, deoxycholic acid; LCA, lithocholic acid; GM, gut microbiota; FGF15, fibroblast growth factor 15; FGFR4, fibroblast growth factor receptor 4; PXR, pregnane X receptor; SHP, small heterodimer partner; GLP-1, glucagon-like peptide-1; TGR5, G protein-coupled receptor.

The signaling pathways activated by bile acids are involved with their receptors, mainly FXR and TGR5. In NAFLD, both receptors are understimulated as deoxycholic acid is decreased [18]. The physiological roles of these two receptors are quite different and as such decreased activation of each receptor causes a different cascade of defects (Figure 1).

FXR is mainly located in the intestines and the liver. The intestinal PXR can promote the secretion of fibroblast growth factor 15/19 (FGF15/19), which enters into the systemic circulation and acts on multiple organ sites [22]. The main organs targeted by FGF15/19 include liver, adipose tissue and muscle. Overexpression of FGF15 in mice promotes decreased fat mass due to increased energy expenditure and thus increased the ability to resist HFD-induced obesity and diabetes [23]. Knockout of *Fgf15* gene in mice accelerated hepatic steatosis in HFD fed mice, with increased expression of PPAR-gamma-2 [24]. A study showed that FGF19 downstream Klotho beta (KLB) was associated with NAFLD [25]. A beta-Klotho mutation rs17618244 G>A caused decreased expression of KLB protein in 249 paediatric patients, which was correlated with the severity of NASH denoted by hepatic ballooning and inflammation [25]. In the cultures of HepG2 and Huh7 cells, reduction of KLB either induced by free fatty acids or the mutation resulted in lipid accumulation and upregulation of lipogenesis enzymes p62, ACOX1, ACSL1 and pro-inflammatory cytokines IL-1beta and TNF-alpha.

FXR in the liver can activate small heterodimer partner 1 (SHP-1), which in turn inhibits CYP7A1 to regulate bile acid synthesis from cholesterol. Therefore, underactivation of FXR in NAFLD leads to increased activity of CYP7A1 and increased synthesis of cholic acid. However, when CYP27A1 levels are decreased, chenodeoxycholic acid synthesis is decreased [26]. FXR is also associated with hepatic steatosis. Activation of FXR decreases fatty acid uptake, fatty acid synthesis and increases fatty acid oxidation [27]. 

Several end organs can activate TGR5. In the intestines, TGR5 activation in L cells increases the secretion of glucogon-like peptide-1 (GLP-1) (Figure 1) [28]. GLP-1 binds to GLP-1R in beta-cells of the pancreas to increase insulin secretion and reduce glucagon synthesis. GLP-1R agonists have been shown to reduce hyperlipidaemia, hypertension and fatty liver in diabetic patients [29]. GLP-1R agonists exenatide, liraglutide and taspoglutide are able to modestly decrease low-density lipoprotein-cholesterol, triglycerides and total cholesterol [30]. FXR has been demonstrated to have co-operative effects with TGR5 in promoting GLP-1 secretion [28]. 

In skeletal muscle and brown adipocyte tissue, TGR5 activation increases energy expenses and insulin sensitivity. Activation of TGR5 results in cAMP-dependent increased activity of thyroid hormone activating enzyme deiodinase 2, leading to increased conversion of T4 to T3, which stimulates energy expenditure [31]. This effect of bile acids is abolished in deiodinase 2 knockout mice, indicating that bile acids are the key mediators of TGR5 activation on T4. In an in vitro cell culture experiment, bile acids increase deiodinase 2 and oxygen consumption in brown adipocytes and skeletal myocytes.

It has been demonstrated that TGR5 activation in macrophages has an anti-inflammatory effect. The production of pro-inflammatory cytokines is reduced through inhibition of NF-kB [32], and inflammasome via PKA ubiquitination of NLRP3 inflammasome. In mice, the knockout of TGR5 has been shown to accelerate LPS-induced inflammation in the liver and abolish the inhibitory effect of TGR5 agonist on the expression of inflammatory mediators [33].

In summary, dysregulation of the bile acid pool in NAFLD can contribute to increased energy expenditure and to a chronic inflammatory status. Dysregulation of bile acids also further disturbs gut dysbiosis and bile acid biosynthesis. Therefore, restoration of the bile acid pool in NAFLD is of significant importance for ameliorating the disease.

### 3.2. Reduced Production of Butyrate

**Figure 2:**
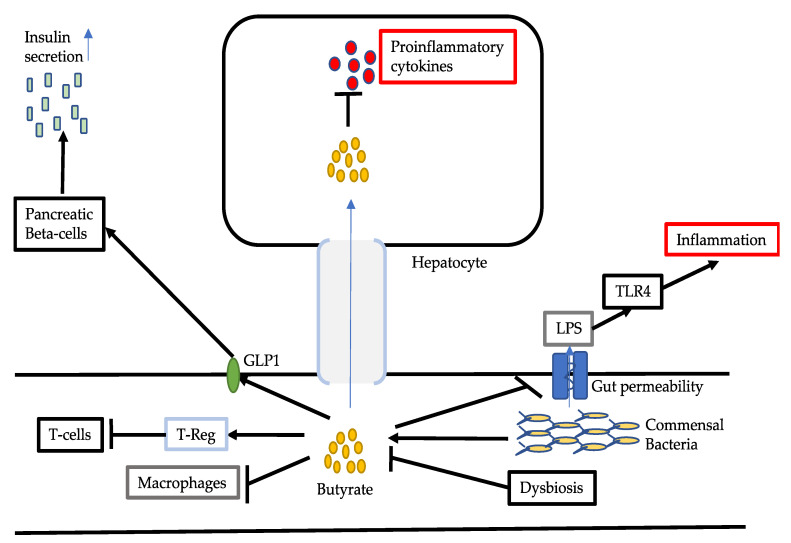
Butyrate in NAFLD. In NAFLD, dysbiosis reduces the levels of butyrate, which is produced by commensal bacteria. Butyrate can reduce intestinal inflammation by activating T-Reg cells, which in turn inhibit T cells. Butyrate also inhibits pro-inflammatory macrophages directly. Through increasing gut barrier, butyrate decreases the entrance of LPS into the blood circulation, thus reduced TLR4-mediated inflammation. By promoting GLP-1 secretion, butyrate stimulates beta-cell to secret insulin. In the liver, butyrate has direct anti-inflammatory effect to reduce pro-inflammatory cytokines. Abbreviations: T-Reg cells, regulatory T-cells; LPS, lipopolysaccharides; TLR4, toll-like receptor 4; GLP-1, glucagon-like peptide-1.

Extensive studies have been conducted reporting that commensal bacterial metabolites such as short-chain fatty acids including acetic acid, propionate and butyrate mediate the regulatory effect on the gut microbiota and on host inflammatory responses. Butyrate is the most potent anti-inflammatory mediator [34,35]. Butyrate can reduce local inflammation in the intestines and prevent the progression of inflammatory responses to the systemic circulation. Butyrate can activate regulatory T cells in the intestines, which in turn inhibits Th17 cells and T cells, thus reducing pro-inflammatory signalling pathways (Figure 2) [34]. Through both HDAC-dependent and -independent manners, butyrate can also inhibit multiple pro-inflammatory signalling pathways [35]. Butyrate can also promote tight junction function and intestinal integrity. Another beneficial effect of butyrate is to provide an energy source to colonocytes to maintain gut health [36]. Alternatively, reduced butyrate levels triggered by gut dysbiosis can result in a low-grade inflammatory status and reduced capability of anti-inflammatory reactions.

Decreased levels of butyrate production in NAFLD could result in increased gut permeability, with increased risk for bacterial and LPS translocation into the systemic circulation (Figure 2). Several recent studies have shown the role of LPS in the pathogenesis of NAFLD and associated mechanisms. Fei et al. (2020) found that the endotoxin-producing bacteria *Enterobacter cloacae* B29, *E. Coli* PY102 and *Klebsiella Pneumoniae* A7 were overgrown in obese NAFLD patients [37]. The administration of B29 to HFD germ-free mice resulted in NAFLD while HFD feeding alone did not cause NAFLD in germ-free mice. The mutation WaaG of B29 that lacks endotoxin did not induce NAFLD. Knockout of the LPS downstream protein TLR4 also abolished the ability of B29 to cause NAFLD. These results suggest that gut dysbiosis could trigger the activation of the LPS-TLR4 axis and contribute to the pathogenesis and NAFLD. Carpino et al. (2020) investigated LPS in the blood and liver from NAFLD patients and found increased levels in both organs with correlation to increased levels of serum zonulin and pNF-kB [38]. Furthermore TLR4^+^ macrophages and TLR4^+^ platelets were increased. In a mouse model of NASH, increased LPS, NF-kB and hepatic inflammation were reduced by inhibition of TLR4 [39]. In HepaRG cells, LPS activated NF-kB, which was inhibited by chemical and small interfering RNA inhibition of TLR4 [39]. In mice fed an HFD, butyrate was reported to decrease LPS-TLR4 axis and thus ameliorate steatohepatitis [40].

Butyrate is capable of upregulating GLP-1R expression to improve NAFLD [41,42]. In NAFLD patients, GLP-1 levels are similar to those from healthy subjects but GLP-1R expression is decreased [41]. In the HFD-fed mouse model, hepatic GLP-1 levels were decreased, and sodium butyrate was reported to increase the expression of GLP-1R and decrease hepatic steatosis. It was also shown that butyrate stimulated GLP-1 expression in HepG2 via HDAC-2 but not GPR43 and GPR109a [41].

Overall, supplementation of sodium butyrate has been shown to prevent simple steatosis progression to steatohepatitis through various mechanisms [43]. Acetate and propionate also have anti-inflammatory effect, however, these two SCFAs can promote lipid accumulation and gluconeogenesis in the liver [44], therefore, they are unlikely to be used for NAFLD therapy.

### 3.3. Amino Acids 

Bacterial metabolites resulting from the metabolism of tryptophan, phenylalanine and tyrosine have been considered to be involved in the pathogenesis of NAFLD. These amino acids and their metabolites from the gut microbiota have been shown to exert various effects on the liver.

#### 3.3.1. Tryptophan

**Figure 3:**
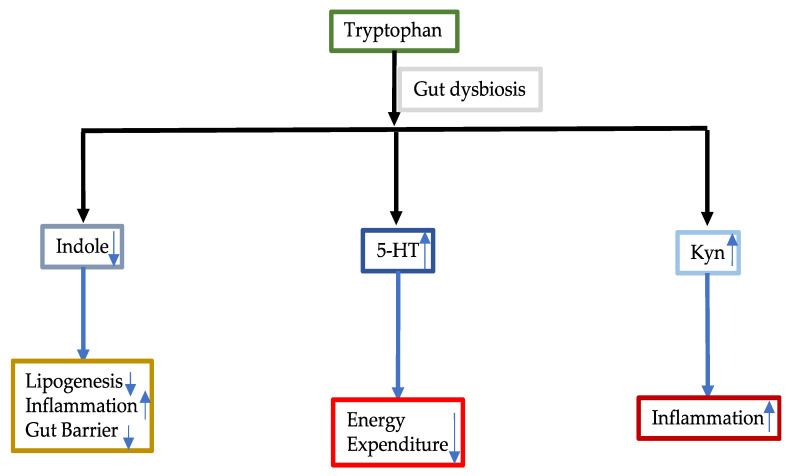
Tryptophan in NAFLD. Gut dysbiosis in NAFLD changes tryptophan metabolisms. Indole can reduce inflammation and lipogenesis and increase gut barrier but it is reduced in NAFLD. Serotonin (5-HT) is increased, which leads to reduced energy expenditure. Kyn (kynurenine) pathway is overactivated, causing inflammation.

Tryptophan is an essential aromatic amino acid, which must be supplied exogenously. There are three metabolic pathways that commensal bacteria in the gut use to metabolize tryptophan (Figure 3) [45]. In the indole pathway, tryptophan is converted into indole which is further metabolized into various derivatives. Through tryptophan hydroxylase 1 (Thp1) and Thp2, tryptophan is converted into serotonin (5-HT). In another pathway, butyrate is catalysed by indoleamine 2,3-dioxygenase (IDO) to produce kynurenines (Kyn). In NAFLD, tryptophan metabolism is disturbed. Supplementation of tryptophan increased intestinal integrity and improved liver steatosis and function in a mouse model of NAFLD established by feeding fructose [46].

The tryptophan metabolite indole was shown to increase intestinal integrity [47]. In germ-free mice, indole production was greatly reduced, resulting in decreased intestinal barrier integrity whereas supplementation with indole increased barrier integrity. This was indicated by increased expression of molecules involved in tight junction and adheres junction structure. In addition, supplementation with indole reduced the severity of colitis that was induced by DDS. In another study, it was shown that circulating indole concentrations were inversely correlated with obesity and liver fat content [48]. Indole reduced the severity of NAFLD caused by an HFD in mice. In cell culture, indole was demonstrated to induce expression of PFKFB3, a result that can promote glycolysis and inhibit inflammation. The role of PFKFB3 in NAFLD was further demonstrated by the disruption of the myeloid PFKFB3 that resulted in more severe hepatic steatosis and inflammation.

The indole derivative indole-3-acetic acid was also reported to reduce hepatic lipogenesis and inflammation in a mouse model induced by an HFD [49]. Indole-3-acetic acid decreased the expression of numerous genes involved in lipogenesis including sterol regulatory element binding-protein 1 (*Srebf1*), stearoyl coenzyme decarboxylase 1 (*Scd1*), peroxisome proliferator-activated receptor gamma (*PPARγ*), acetyl-CoA carboxylase 1 (*Acaca*) and glycerol-3-phosphate acyltransferase, mitochondrial (*Gpam*). Indole-3-acetic acid also reduced fasting blood glucose levels and improved insulin resistance as well as macrophage infiltration and expression of MCP-1 and TNF-alpha.

Serotonin has also been associated with NAFLD. Increased 5-HT was shown to inhibit energy expenditure of brown adipose tissue through blocking mitochondrial uncoupling protein 1 [50]. In *Thp1*^−^ deficient mice, high-fat diet feeding failed to cause obesity, insulin resistance and NAFLD. Small molecular inhibitors of Thp1 produced a similar effect, blunting high-fat-diet-induced NAFLD. Choi et al. (2018) demonstrated that knockout of *htr2a* gene (liver serotonin receptor 2 a) had the same effect as knock out of *Thp1* in reducing high-fat-diet-induced hepatic steatosis [51]. Inhibition of HTR2a receptor had also reduced hepatic steatosis induced by feeding an HFD to mice.

Kyn pathway is overactivated in NAFLD [52]. Inflammatory factors IFN-gamma, IL-1, LPS and TNF-alpha stimulate the expression of IDO, which is the rate-limiting enzyme of the Kyn pathway [53,54,55]. Kyn can cause inflammation in various organs and Kyn/Trp has been used as an indicator of the progression of inflammation [56,57,58].

#### 3.3.2. Phenylalanine

Phenylalanine (PA) and its derivative phenylacetic acid (PAA) have been shown to participate in the pathogenesis of NAFLD. Hoyles et al. (2018) showed that transplantation of a steatosis-associated microbiome into mice increased hepatic triglycerides and circulating branched chain amino acids and trimethylamine N-oxide (TMAO) [16]. Metagenomic analysis showed increased abundance of microbial gene pathways associated with PAA production, whereas metabolomic analysis showed a strong association of PAA with steatosis. In the primary hepatocyte cell culture, PAA and PA produced a synergistic effect in triglyceride accumulation. The expression of many lipogenesis genes was altered. PAA inhibited pAkt, thus decreasing insulin biochemical functions. In mice, chronic treatment of PAA caused hepatic steatosis [16]. Two weeks of PAA treatment resulted in increased hepatic triglycerides and decreased isoleucine exclusion. This result indicates the increased use of BCAA for lipid accumulation.

#### 3.3.3. Branched Chain Amino Acids

Some branched chain amino acids including valine, leucine and isoleucine are associated with NAFLD. Sunny et al. (2015) demonstrated that impairment of TCA by BCAA was associated with mitochondrial dysfunction in NAFLD [59]. Gaggini et al. (2018) showed that the severity of NAFLD, examined at biopsy, indicated by ballooning and/or inflammation of the liver was associated with increased BCAA [60]. BCAA can cause insulin resistance which is associated with obesity and T2DM [61,62]. In 277 insulin resistant nondiabetic Danish individuals, Pedersen et al. (2016) found that increased BCAA levels were consistent with enhanced biosynthetic potential for BCAAs by the gut microbiota [63].

### 3.4. Choline and Methionine

Choline can be converted by the gut microbiota into trimethylamine (TMA) directly or via betaine, and TMA is oxidized into trimethylamine N-oxide (TMAO). In NAFLD, the conversion of choline into TMA/TMAO are increased, leading to deficiency of choline and accumulation of TMA/TMAO [64,65]. Choline can promote very low-density lipoprotein transportation out of the liver and deficiency of choline results in accumulation of lipids in the liver. In animal models, the deficiency of choline has been shown to cause NAFLD. Arao et al. (2020) used a methionine/choline-deficient diet to establish a NASH model and found that mitochondrial DNA content was decreased [64]. Hernandez et al. (2020) reported that high fructose-, fat- and cholesterol feeding in pigs caused hepatic steatosis and inflammation, with impaired FXR-FGF19 signaling [65]. The severity of NAFLD in this model was associated with increased choline metabolites.

### 3.5. Ethanol

Ethanol is normally produced in small amounts in the intestines and metabolized in the liver by alcohol dehydrogenases. Excessive ethanol can cause fluctuations in redox potential changes intracellularly that lead to increased inflammatory responses. Interestingly, ethanol has been recognized as a participant in the pathogenesis of NAFLD. As early as in 2000, Cope et al. proposed that blood levels of ethanol were associated with changes in the gut microbiota [66]. Subsequent studies have provided additional evidence and associated mechanisms.

Dysbiosis in NAFLD has been associated with bacteria such as *Escherichia*, *Bacteroides*, *Bifidobacterium*, *Clostridium* and *Klebsiella pneumonia* that can produce alcohol [67,68,69]. Zhu et al. (2013) studied the endogenous production of ethanol in the gut in NAFLD and observed high increased ethanol levels in NASH patients compared to healthy subjects or in obese non-NASH patients [67]. In these NASH patients, amount of alcohol-producing bacteria of genus *Escherichia* from Proteobacteria was five times higher. Yuan et al. (2019) demonstrated that *Klebsiella pneumonia* K14, which produces high amounts of alcohol, was causal for NAFLD [68]. Transfer of clinical isolates of the bacterium from patients into mice caused NAFLD-like changes. Chen et al. (2020) introduced the bacteria with a mutation K14-adh, which produced higher alcohol levels and another mutation K14-Δadh, which produced less alcohol [69]. It was revealed that K14-adh caused the highest mitochondrial damage, higher than K14 while K14-Δadh caused a milder injury than K14.

Engstler et al. (2016) demonstrated an additional mechanism for increased blood levels of ethanol and showed that insulin resistance caused an abnormal ethanol metabolism [70]. In children diagnosed with NAFLD, dietary patterns and intestinal bacterial growth were similar to healthy subjects, yet plasma ethanol levels were higher. There were no differences in ethanol levels in portal vein and chyme from the gastrointestinal tract in ob/ob and control mice but that in the vena cava were different. Alcohol dehydrogenase was lower in ob/ob mice, and anti-TNF reduced ethanol levels. Given the contentious nature of the reported data, further studies are needed to clarify the roles of ethanol in different types of NAFLD.

## 4. Modulation of Gut Microbiota and Its Metabolites

In view of the important role of dysbiosis of the gut microbiota in the pathogenesis of NAFLD, important efforts have been progressed to explore approaches that could correct gut microbiota dysbiotic profiles and the metabolites elaborated. These approaches have included the administration of probiotics, prebiotics and synbiotics and the use of FMT and FMD. These interventional modalities have been extensively studied in animal models with promising outcomes, as well as in clinical trials.

### 4.1. Probiotics

Various probiotic bacteria have been tested in animal models of NAFLD. The results obtained are promising [71], which become the basis for clinical studies in humans. The most used probiotics are from genera *Lactobacillus*, *Bifidobacteria* and others such as *Streptococcus thermophilus and the Saccharomyces yeast*. Recently, butyrate-producing bacteria have also been highly regarded. 

#### Lactobacillus

Several studies showed that *Lactobacillus casei* strain Shirota (Lcs) was effective in NAFLD. Okubo et al. (2013) revealed that administration of Lcs increased not only Lcs in the gut but also other lactic bacteria in methionine-choline deficient NASH mice [72]. The gut inflammation and serum LPS concentrations were reduced, and hepatic parameters improved including decreased inflammation and fibrosis. Naito et al. (2011) demonstrated that Lcs increased insulin sensitivity, reduced blood levels of glucose and increased LPS-binding protein, which indicates improved endotoxemia in diet-induced, db/db, ob/ob and KK-A(y) mouse obesity models [73]. In a fructose-diet mouse model, Lcs reduced hepatic steatosis, ALT [74]. The associated mechanisms were identified to be inhibition of TLR4 signalling and upregulation of PPAR-gamma [74].

*Lactobacillus plantarum* has been revealed to be effective in ameliorating NAFLD. Zhao et al. (2020) reported that *Lactobacillus plantarum NA136* reduced severity of NAFLD in a mouse model established by a high-fat/high-fructose diet [75]. The probiotics changed the gut microbiota with an increase in *Bifidobacteria*. The gut barrier was increased, inflammation reduced and insulin sensitivity increased. Nguyen et al. showed that *L. plantarum* PH04 reduced cholesterol and triglycerides with increased intestinal BSH activity [76]. Wang et al. showed that *L. plantarum* MA2 reduced both blood and hepatic levels of cholesterol and triglycerides with increased lactic acid bacteria and *Bifidobacteria* [77]. Li et al. (2014) also showed that *L. plantarum* NCU116 reduced hepatic fat accumulation and improved liver function as well as decreased endotoxemia in a high-fat-diet-induced NAFLD model. In a rat model of NAFLD induced by high-fat/high-fructose diet, Park et al. (2020) showed that *Lactobacillus plantarum* strains ATG-K2 and ATG-K6 reduced body weight gain, decreased hepatic lipid accumulation, alanine aminotransferase and aspartate transaminase and lower lipogenesis gene expression [78]. In faecal samples, there were higher proportions of Bacteroidetes and lower Firmicutes.

Another probiotic species—*Lactobacillus rhamnosus GG* (LGG) has also attracted significant research attention. Ritze et al. (2014) demonstrated that LGG administration reduced gut inflammation by inhibiting NF-kB signalling pathway in a model of NAFLD with the mice fed on a fructose diet [79]. The blood LPS levels were reduced due to increased gut barrier function. LGG decreased hepatic inflammation indicated by decreased TNF-alpha, IL-8R, IL-1beta mRNAs and hepatic fat accumulation. Kim et al. (2016) found that LGG reduced hepatic fat accumulation and hepatic inflammation [80]. LGG also reduced liver weight and mesenteric and subcutaneous adipose tissues. Liu et al. found that the probiotic culture supernatants of LGG reduced steatosis and inflammation with increased butyrate production in a mouse NAFLD model established by high-fat/high-fructose diet and intermittent hypoxia exposure [81].

Other Lactobacillus species such as *acidophilus L1, paracasei, johnsonii BS15, leuteri GMNL-263* and *gasseri BNR17* have also been shown to have beneficial effects on NAFLD [82,83,84,85,86]. Although additional strains have been tested, studies to select an optimal set of probiotic strains is as yet undefined. Recently, Naudin et al. (2020) reported that *Lactococcus lactis* subsp. cremoris reduced high-fat-diet-caused weight gain, hepatic steatosis and inflammation as well as blood levels of cholesterol and glucose intolerance compared with LGG [87].

There are currently no clinical trials that have reported robust outcomes. A recent meta-analysis from 1555 NAFLD patients showed that administration of probiotics had beneficial effects on BMI, alanine aminotransferase, aspartate transaminase, gamma-glutamyl transpeptidase, insulin, homeostasis model assessment-insulin resistance and total cholesterol but not on fasting blood sugar, lipid profiles and TNF-alpha [88]. 

Numerous studies have been made to use combination of multiple bacteria to improve efficacy of probiotics. VSL#3 is a probiotic, which is composed of 8 bacterial strains that include *Bifidobacteria* (*B. breve*, *B. longum* and *B. infantis*), *Streptococcus thermophilus*, *L. plantarum*, *L. acidophilus*, *L. paracasei* and *L. delbrueckii* subsp. *bulgaricus*). VSL#3 has been studied both preclinically and clinically, showing its ability to inhibit NF-kB, reduce hepatic inflammation, reduce hepatic fat accumulation and increase insulin sensitivity [89,90]. Mei et al. (2015) also tested a probiotic with combined bacteria (LGG, *Lactobacillus plantarum* WCFS1 and anthraquinone from *Cassia obtusifolia* L.) [91]. The formulation has been reported to improve insulin sensitivity, reduce serum levels of lipids and increased intestinal barrier. Xue et al. (2017) demonstrated that a probiotic with mixed bacteria of *Bifidobacterium infantis*, *Lactobacillus acidophilus,* and *Bacillus cereus* reduced NAFLD liver pathological changes by inhibiting LPS/TLR4 axis [92]. Kim et al. (2017) showed that a probiotic beverage, Kefir, postulated to contain more than 50 species of lactic acid bacteria and yeast increased gut barrier and reduced fat accumulation in the liver through upregulation of PPAR-alpha gene. The blood levels of IL-6 were also markedly reduced [93]. The combination of multiple strains of commensal bacteria in one probiotic produces strong beneficial effects in animal NAFLD experiments. These observations may be related to the various mechanisms exerted by various bacteria. The pathogenic factors that lead to the development of NAFLD involve bile acids, butyrate, amino acids, choline and ethanol. However, systemic investigations relevant to the mechanisms of the probiotics composed of a mixture of bacteria has not been done and warrants sufficient studies.

### 4.2. Prebiotics

Prebiotics are defined as nondigestible food ingredients in the diet but that are subject to fermentation by gut microbiota. Many studies have shown that various prebiotics can reduce NAFLD pathogenesis. However, their effects on bile acid metabolism and butyrate production have not been well focused. For example, fructooligosaccharides (FOS) and inulin have been shown to improve NAFLD in various animal models. However, the mechanisms are not well understood relative to the changes that have been described for altered bacterial metabolites.

Fructooligosaccharides (FOS) are composed of short fructose chains of various oligofructans with different lengths of chains. FOS have been tested in animal models of NAFLD established by administration of monosodium glutamate, methionine-choline deficiency or with feeding of a high-fat diet. FOS have been reported to ameliorate NAFLD parameters such as steatosis and inflammation in animal models of NAFLD [94,95]. 

In monosodium glutamate-injected mice, Takai et al. (2020) showed that supplementation with FOS reduced hepatic steatosis, inflammatory cell infiltration and hepatocyte ballooning as well as decreased expression of fatty acid synthase and glycerol-3-phosphate acyltransferase [94]. This has been associated with increased faecal SCFAs including butyrate, propionate and acetate. In addition, serum propionate was also shown to be increased by more than twofold. Kok et al. (1996) demonstrated that feeding FOS could reduce lipogenesis, and thus resist fructose-increased triacylglycerol very low-density lipoprotein (VLDL) and fatty acid synthase and phosphatidate phosphohydrolase [95]. Delzenne et al. (2001) reported that FOS decreased triacylglycerol in VLDL in rats with decreased gene expression of fatty acid synthesis enzymes including acetyl-CoA carboxylase, fatty acid synthase, malic enzyme, ATP citrate lyase and glucose-6-phosphate dehydrogenase [96]. The administration of FOS reduced postprandial insulin and glucose levels. In methionine-choline-deficient mice, FOS decreased hepatic steatosis, inflammation and Kuffer cells [97]. Decreased TLR4 could indicate reduced endotoxemia. The faecal butyrate and faecal IgA were increased. The intestinal microbiota was also improved. Moreover, it has been reported that FOS reduced liver fat content [98]. In the animal model of NAFLD, induced by 70% sucrose, treatment of FOS reduced hepatic and cardiac steatosis.

Inulin is a special type of fructan, which is characterized by beta-2:1 bonds. It can be fermented by gut bacteria to produce butyrate. Chong et al. (2020) reported that supplementation of inulin further reduced ALT induced by metronidazole in a randomized double-blind placebo-controlled clinical trial [99]. Weitkunat et al. (2015) reported that inulin feeding to mice increased production of SCFAs and reduced expression of genes involved in lipogenesis and fatty acid elongation/desaturation [100].

These data showed that FOS improved NAFLD through the restoration of the gut microbiota. The mechanisms could be associated with increased butyrate production, decreased endotoxemia and decreased lipogenesis. However, what effects they may have on bile acids, ethanol and amino acids are yet to be elucidated.

### 4.3. Synbiotics

A synbiotic is the combination of probiotic bacteria with a prebiotic in a formulation and as such has the advantage of producing increased levels of butyrate through the action of the probiotic bacteria that provide materials for commensal bacteria to metabolize the prebiotic. Liu et al. (2019) showed that probiotics and synbiotics are effective in NAFLD treatment in a meta-analysis with 782 patients [101]. 

In a rat model of NAFLD using a high-fructose diet, a synbiotic formulation made up *Lactobacillus fermentum* CECT5716 and FOS, reduced liver steatosis and insulin resistance [102]. Yao and colleagues used an HFD to induce NAFLD in mice and treated with *Lactobacillus paracasei* N1115, FOS and combination [103]. All three treatments reduced steatosis with the combination having the highest effect. Alves et al. showed that a synbiotic reduced steatosis via increased lipid beta oxidation indicated by the upregulation of PPAR-alpha and decreased lipogenesis indicated by SREBP-1c and FAS [104].

In NASH patients, a synbiotic composed of *Bifidobacterium longum* and FOS reduced AST, cholesterol, CRP, TNF-alpha, HOMA-IR, serum endotoxin, steatosis and NASH activity index [105]. A phase II clinical trial investigated the effect of a synbiotic composed of FOS and *Bifidobacterium animalis* subspecies lactis BB-12 in 104 NAFLD patients [106]. The synbiotic increased faecal *Bifidobacterium* and *Faecalibacterium* and decreased *Oscillibacter* and *Alistipes* species, whereas placebo did not cause these changes. The synbiotic, however, did not lower liver fat and fibrosis more than placebo. This is an important outcome to consider in the future selection of NAFLD patients for the clinical investigations with probiotics and or synbiotic formulations. 

### 4.4. FMT

FMT was introduced by Eiseman and colleagues in 1958 for the treatment of pseudomembranous colitis [107]. Later, it was successfully applied in the treatment of *Clostridium difficile* infections. Currently, it is being studied for the treatment of NAFLD. Compared to probiotics and synbiotics, FMT can provide a wide range of commensal bacteria as well as other microbials which facilitate the maintenance of gut microbial ecology. Both animal studies and clinical trials have shown promising outcomes for the FMT application in NAFLD.

In an animal model of NAFLD, Zhou et al. (2017) set up three groups of mice including a control, an HFD and an HFD plus FMT group to demonstrate the beneficial effects of FMT [108]. It was reported that FMT from control mice reduced HFD-induced steatohepatitis with decreased hepatic lipid accumulation, decreased pro-inflammatory cytokines IL-17 and increased anti-inflammatory cytokines IL-4 and IL-22 as well as increased intestinal beneficial bacteria *Christensenellaceae* and *Lactobacillus.* Additionally, increased levels of intestinal butyrate, tight junction protein ZO-1 and decreased blood endotoxemia were reported. 

Vrieze et al. (2012) reported the results from a clinical trial [109]. It demonstrated that FMT from healthy lean individuals to patients with metabolic syndrome increased insulin sensitivity. It also improved the intestinal microbiota with increased intestinal butyrate-producing bacteria [109]. Several trials to test FMT in NAFLD patients are currently ongoing (NCT04465032, NCT02469272 and NCT02721264).

### 4.5. FMD

It has been advanced that a 10% reduction in body weight is an effective treatment in reducing the burden of NAFLD. Therefore, FMD is a beneficial strategy in NAFLD. FMD makes calorie restriction an easier and achievable outcome by intaking food that mimics fasting. The FMD is composed of low-calorie nutrients, sugars and proteins and is high in unsaturated fatty acids [110]. Low calorie diets have been studied in rats together with polysaccharides to evoke a beneficial effect on NAFLD [111]. It has also been shown to increase beta-cell regeneration, insulin restoration and glucose profiles in type 1 and type 2 diabetes in mice [112,113]. In mice, FMD has been shown to restore the gut microbiota and bacterial metabolites [113,114].

Wei et al. (2017) randomized 100 healthy subjects to FMD or placebo for three-months with FMD subjects having 5 consecutive days of FMD. The study reported decreased BMI, blood pressure, total body fat and IGF-1 [110]. In NAFLD patients, calorie restriction has been shown to reduce body weight, BMI and improved lipid profiles as well as liver function indicated by ALT and AST compared to resveratrol and placebo groups [115]. How FMD affects the gut microbiota and bacterial metabolites in humans is not well elucidated. As the data are still limited in clinical trials, further studies are warranted [116].

## 5. Conclusions

Gut dysbiosis has been associated with NAFLD. Different stages of NAFLD have different signatures associated with the intestinal microbiota. The effects of an altered gut microbiota in abundance and diversity are mediated by many bacterial metabolites including bile acids, butyrate, choline, amino acids and ethanol. Modulation of the intestinal microbiota and supplementation of some bacterial metabolites may have a therapeutic benefit. This has been demonstrated in animal models of NAFLD. Current clinical studies are at a nascent stage.
